# Genomicus update 2015: KaryoView and MatrixView provide a genome-wide perspective to multispecies comparative genomics

**DOI:** 10.1093/nar/gku1112

**Published:** 2014-11-06

**Authors:** Alexandra Louis, Nga Thi Thuy Nguyen, Matthieu Muffato, Hugues Roest Crollius

**Affiliations:** 1 Ecole Normale Supérieure, Institut de Biologie de l'ENS, IBENS, Paris F-75005, France; 2 Inserm U1024, Paris F-75005, France; 3 CNRS, UMR 8197, Paris F-75005, France; 4 European Molecular Biology Laboratory, European Bioinformatics Institute, Wellcome Trust Genome Campus, Hinxton, Cambridge CB10 1SD, UK

## Abstract

The Genomicus web server (http://www.genomicus.biologie.ens.fr/genomicus) is a visualization tool allowing comparative genomics in four different phyla (Vertebrate, Fungi, Metazoan and Plants). It provides access to genomic information from extant species, as well as ancestral gene content and gene order for vertebrates and flowering plants. Here we present the new features available for vertebrate genome with a focus on new graphical tools. The interface to enter the database has been improved, two pairwise genome comparison tools are now available (KaryoView and MatrixView) and the multiple genome comparison tools (PhyloView and AlignView) propose three new kinds of representation and a more intuitive menu. These new developments have been implemented for Genomicus portal dedicated to vertebrates. This allows the analysis of 68 extant animal genomes, as well as 58 ancestral reconstructed genomes. The Genomicus server also provides access to ancestral gene orders, to facilitate evolutionary and comparative genomics studies, as well as computationally predicted regulatory interactions, thanks to the representation of conserved non-coding elements with their putative gene targets.

## INTRODUCTION

The rise of high-throughput DNA sequencing has revolutionized the field of genomics by creating an abundance of genome information in all domains of life. This volume and diversity of data enables ever more powerful evolutionary studies aimed at understanding past genomic events, and generally, to reconstruct the History of life, including the genomic characteristics of the common ancestors of extant species. Advanced tools facilitating the intuitive manipulation of such complex data are thus needed, because the interpretation of specific evolutionary events often requires a broad evolutionary perspective that includes deep branches and many species. Toward this end, we continue to develop, maintain and improve the Genomicus server to offer innovative ways to access genomes data. The Genomicus software is a dynamic visualization interface that enables comparisons between potentially unlimited numbers of genomes. It provides a comprehensive overview of genome organization between modern species and in the case of vertebrates and flowering plants, Genomicus also provides extensive information on reconstructed ancestral genomic organization. Users can intuitively query and navigate within and between genomes in a phylogenetic context to examine the evolution and the conservation of a specific locus in a species of interest. Navigation in Genomicus can be performed in three dimensions: linearly along the axes and the position of chromosomal genes, transversally between different species and chronologically along an evolutionary axis. For 4 years now, Genomicus is a web server that facilitates comparative genomics projects across a broad phylogenetic range.

Since our previous NAR report (1), we improved Genomicus in two directions. First, we enhanced the ergonomy and rationality of internal menus, and we provide a major new entry point to the database by allowing BLASTP searches with a protein sequence. Second, we developed two new interactive graphical views for pairwise genome comparisons (KaryoView and MatrixView), and extended existing views with more advanced features.

## DATA SOURCES AND COMPUTATION

### Data sources

All of the data about extant genes that are available on the Genomicus web servers come from the Ensembl (2) and EnsemblGenome (3) databases. The current (November 2014) Genomicus server dedicated to vertebrates is more specifically based on Ensembl release 76, which contains genome annotations for 68 extant species. From Ensembl, we have downloaded all the protein-coding sequences together with their gene location, the gene family they belong to, the percentage of similarity between pairs of proteins and the computed dN/dS ratio (the ratio of non-synonymous to synonymous mutations in coding sequences, an indicator of the rate of sequence diversification).

### Ancestral genome inference

Ancestral gene content and gene order are inferred by a method called AGORA (Algorithm for Gene Order Reconstruction in Ancestor) (4). Ancestral gene contents are inferred from the gene trees (computed and reconciled with the species tree) provided by EnsemblCompara (5). Briefly, the inference of the ancestral gene order is deduced from pairwise genome comparisons of all the pairs of extant genomes whose evolutionary path includes the ancestor. For instance, the human-dog comparison is used by AGORA to reconstruct the gene order of their last common ancestor (*Boreoeutheria*), but also other ancestral species on the evolutionary path such as *Primates*, *Hominidae*, *Carnivora* and *Canidae*. The common adjacencies are recorded and combined in a weighted graph where nodes represent genes and link between nodes the number of time the adjacency is conserved. The graph is then processed using a top-down greedy algorithm where the links of highest weight are selected first and are used to select the most likely gene-to-gene adjacency in case of multiple choices and while avoiding cycles. The algorithm ends with a fragmented genome composed of linear paths for each ancestor (that we call contigs). After this first step, a second round of AGORA is performed, by considering the contigs instead of genes as unit of comparison. The algorithm then compares adjacencies of contigs in each pairwise extant genome that is informative for an ancestor, and builds a graph of contig adjacencies. The graph is then linearized to obtain final blocks (or scaffold) of ancestral gene order.

### Conserved non-coding element detection and gene target prediction

For vertebrates, conserved non-coding elements (CNEs) and their potential target genes are available in Genomicus. CNEs are computed from multiple alignments between 46 vertebrate genomes projected on the human genome, generated using Multiz and other tools from the UCSC (University of California, Santa Cruz) and Penn State Bioinformatics groups, and made available on the UCSC website (6). An algorithm named ScanMaf (M. Naville et al., submitted for publication) scans the alignment and looks for conserved regions of a minimal length (10 bp) and identity (90%) and extends them by accepting up to three non-conserved columns on each side. This algorithm does not require a fixed set of key species in the alignment, but instead a minimal number of eight species. The CNEs displayed in Genomicus have a minimal size of 20 bp. CNEs are excluded from regions overlapping protein-coding sequences in all of the species considered. Once the CNEs are detected, they are considered as potential enhancer. A scoring method to identify evolutionary conservation of linkage between CNEs and neighboring genes has been developed, to define potential target genes (Naville, M. *et al*, submitted). CNEs are named according to the following nomenclature: RegHsaxxxxxxx, where Reg stands for regulatory, Hsa for the species where they have been identified (here, Homo sapiens) and x-s represents a unique numeric identifier.

## GENOMICUS VIEWS

### Improved queries, top menu features

**Figure 1.**
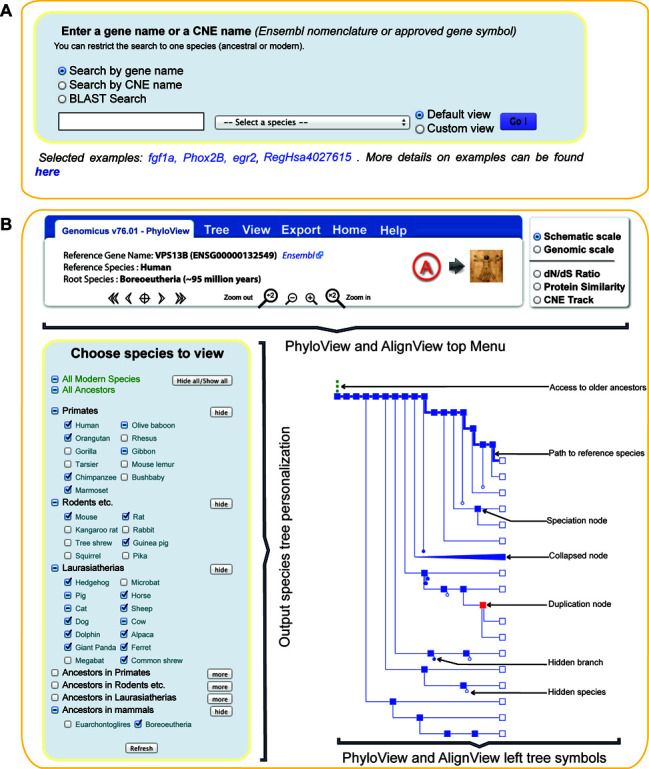
Main menus of the Genomicus server. (**A**) The Genomicus entry point is a gene name such as an HGNC symbol, an Ensembl gene ID or a free text search against the gene descriptions. CNE can be queried by name, and even raw protein sequences can be searched with BLASTP against all the proteins stored in the Genomicus database. Users can restrict their search by selecting a species of interest by the dropdown menu. (**B**) This figure describes the common parts of the two multiple genome comparison views, AlignView and PhyloView. Users can choose the species (extant and ancestral) that are displayed by (un)ticking them in the foldable list available on the left of the page. The last part of the figure explains all the symbols used in the species or gene trees drawn in Genomicus.

Based on usage survey and suggestions from users, querying Genomicus has been improved (Figure 1A). The standard query interface accessible using a gene name, HGNC symbol or an Ensembl gene ID remains as before, but has been extended to include free text to be matched to Ensembl gene descriptions without loss in response time. CNEs can also be queried by names using their nomenclature (RegHsaxxxxxxx). Context-sensitive name completion is now in place, and is activated when the user has entered at least three letters. The second major change is the possibility to query the server with a protein sequence, through a BLASTP search. On the home page, users can paste in a protein sequence, select a query database and compare the sequence against all the proteins available in the chosen database. This new mode of access is useful when users start from a gene/protein that is not in a gene family stored in Genomicus, e.g. because the genome has not yet been sequenced. The best HSP (High-scoring Segment Pairs) will be displayed to allow the user to choose the gene of interest that most closely resembles their starting sequence of interest. This selected match is then considered as a ‘reference gene’, which belongs to a ‘reference species’. This ‘reference gene’ is the starting point to explore its genomic context and its evolution.

From there, the default view is PhyloView, which compares gene order on both sides of a reference gene in all genomes that possess a homolog of this gene. The other view is AlignView, which aligns a reference genome to other genomes in the neighborhood of the reference gene, even to genomes that do not possess a reference homolog gene as long as they possess at least two orthologs of neighboring genes on the same chromosome or scaffold. Both views have been previously described in (1)).

In PhyloView and AlignView, the top menu has been updated to be more intuitive and easy to use (Figure 1B). The main menu still includes information on the reference gene as well as navigation tools. The top menu combines information on the reference gene and species, with external links. The « Tree » sub-menu gives options to configure the output content (hide all low coverage genomes or ancestors, focus on paralogs…), while the « View » sub-menu proposes links to the other Genomicus views: PhyloView and AlignView of the same reference gene, MatrixView or KaryoView with the same reference species. The « Export » sub-menu can be used to export SVG or text files of the output for subsequent editing and figure preparation. The second rectangle (right of the main menu) is a new configuration box to switch between the two horizontal scales (top half), and between the property that is used to color genes (bottom half). A new functionality is available to select extant and ancestral genomes to be shown. The standard method consists in clicking on individual tree nodes that users want to hide or collapse. This still exists, but Genomicus now features a foldable overview of all extant and ancestral species, which is more convenient to configure the display in a global fashion.

### Multiple genome comparison: update of AlignView and PhyloView displays

**Figure 2.**
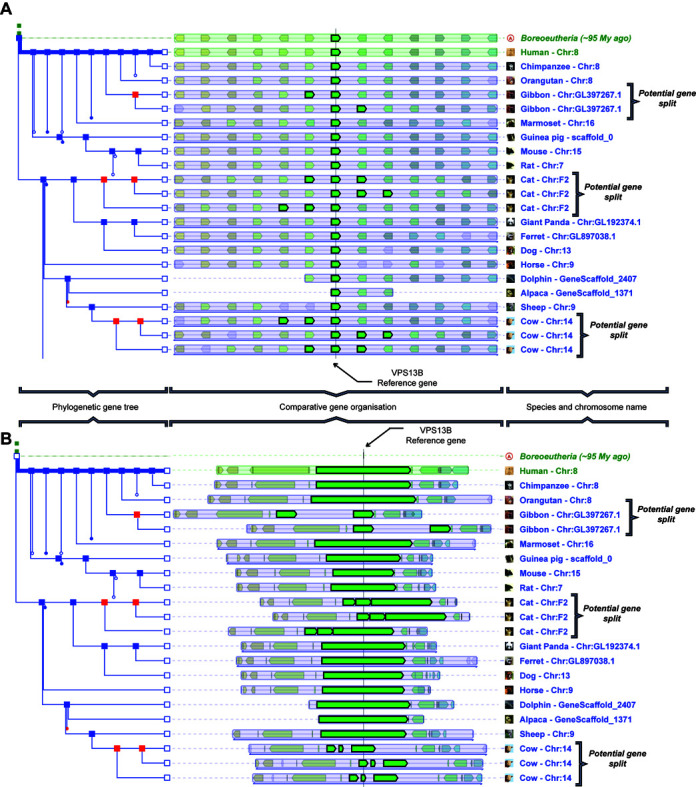
PhyloView representation of the VSP13 reference gene in the human genome. (**A**) In the PhyloView schematic scale representation, mouse-hovering the reference gene VSP13 highlights all its homologs in the other species. Orthologs of VSP13 in gibbon, cat or cow have co-located paralogs that can either be true species-specific paralogs, or spuriously annotated genes in these species. (**B**) The genomic scale view of the same PhyloView page gives more support to the latter hypothesis since the total genomic span of those paralogs is similar to the length of all VSP13 orthologs. This genomic-scale display could then help annotators to detect potential gene splits.

The aim of the Genomicus web server is to provide a way to explore spatial information related to gene organization within and between genomes. The two multiple genome comparison views, PhyloView and AlignView, available since 2010, allow the study of gene and genome evolution with schematic display of genes. Gene positions are defined according to the shortest transcripts of every gene (to minimize cases of overlapping genes). Orthology and paralogy information is shown through colors (Figure 2A).

One of the advantages of comparative genomics is to facilitate genome annotation. Typically, predicted proteins from one genome are ‘projected’ to another genome through sequence comparisons. However, the target genome may not be assembled correctly at the position of a given gene, and this artefact may go unnoticed until a locus-specific analysis is carried out. New features in Genomicus may now greatly facilitate this process. In PhyloView, the user can now switch from the traditional schematic scale representation of genes to a new ‘genomic scale’ using the top right menu of the page, where genes are drawn using their real genomic coordinates. In this display, the longest genomic fragment shown at any time is automatically set to a width of 1000 pixels, and other genomes are scaled accordingly. In the example described in Figure 2, the importance of being able to switch between the two views is underlined by showing cases where potential gene duplications in the schematic display of PhyloView can clearly be dismissed as annotation artefacts in the new ‘genomic scale’ view (Figure 2B).

**Figure 3.**
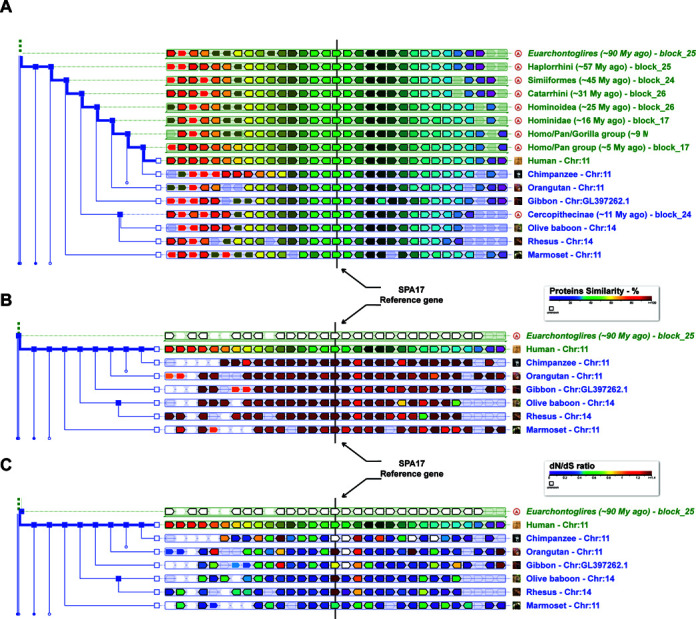
PhyloView example centered on the SPA17 gene of human. (**A**) Default view of PhyloView in the schematic scale. The SPA17 gene (reference gene) and all its homologs are centered on the middle line. The thick blue line in the specie tree leads the root ancestor to the reference species (*Homo sapiens*) and other species. One can switch between the default view (based on gene tree membership) and representation of similarity score between proteins of the same family (**B**) or dN/dS ratio comparisons (**C**). Those two alternative representations highlight that despite the fact that SPA17 shares a very high similarity score (almost 100% of identity) to all its orthologs, selection patterns are widely different (as suggested by dN/dS).

The second new feature (Figure 3), available in both PhyloView and AlignView, is the ability to use the colors of genes to represent the percentage of sequence identity between homologs of the reference genome instead of orthology (Figure 3B). A second new option is to show the dN/dS ratio computed from multiple alignments of coding sequences from genes of the same family (Figure 3C).

By right-clicking on the species name (right-hand side), users can now also switch to other representations of the comparison of the reference genome and the genome that has been selected.

### CNE display

CNEs located in the intron of genes or in intergenic space, and computed from the 46-way UCSC multiple alignment, have been a regular feature of past Genomicus releases. Now, CNEs have been individually named using a simple nomenclature starting with the prefix ‘Reg’ followed by a three-letter species identifier (e.g. Hsa for human) and a seven-digit number. The names are permanent, will be carried over in successive releases and can be used to directly query the database, as for genes. Alternatively, to view all human CNEs of a given locus at once, the PhyloView display has a ‘CNE track’ option on the top right menu. By convention, because CNEs are always plotted in the schematic scale display, intronic CNEs are displayed on the lower half of the right-hand intergenic space of the gene in which they are included.

Using the ‘mouse-over’ function over a CNE symbol, a neighboring gene may be highlighted. In such case, the CNE is a predicted regulatory enhancer, and the highlighted gene is a predicted target of this enhancer. The method used to predict these interactions relies on an evolutionary linkage score between the CNE and its most probable target (Naville, M. *et al*, submitted). Only CNEs showing a linkage score higher than 0.9 to their target human gene are displayed in Genomicus.

### Pairwise genome comparison: KaryoView and MatrixView

**Figure 4.**
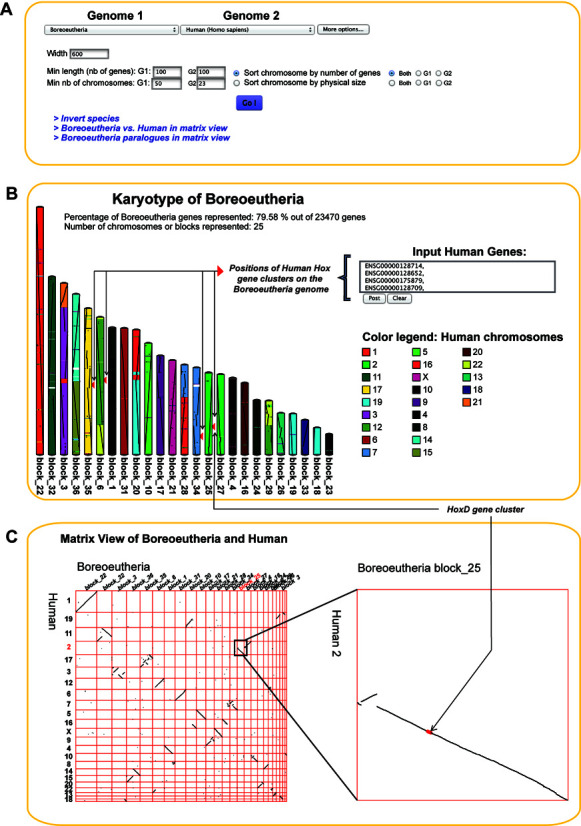
KaryoView and MatrixView of the reconstructed boreoeutherian genome versus *Homo sapiens*. (**A**) The menu to select the two genomes (‘Genome 1’ and ‘Genome 2’) is the same in both views. (**B**) KaryoView allows users to visualize a genome (extant or ancestral) according to the colors of the syntenic chromosomes of a different genome (extant or ancestral). (**C**) MatrixView is a dot-plot comparison of two genomes (here *Homo sapiens* against its ancestor *Boreoeutheria*). Users can click cells of the matrix to zoom in the comparison. At this higher zoom level, individual genes are clickable and linked to PhyloView. Here, when selecting the boreoeutherian scaffold *block_25* versus the human chromosome 2, the previously-queried HOXD genes appear in red.

With PhyloView and AlignView, Genomicus provides an intuitive way to compare extant and ancestral genomes in a local gene environment. Two new graphical representations, called KaryoView and MatrixView (Figure 4), are now available to allow a more global view of the comparison. Figure 4 describes the comparison of the ancestral reconstruction of the boreoeutherian genome with *Homo sapiens* chromosomes. Both views interactively display chromosome-scale synteny between any pair of genomes (extant and ancestral ones). The top menu is the same in KaryoView and MatrixView (Figure 4A). Hyperlinks give quick ways of switching between the two different views, inverting the two selected species, and switching to a dot-plot self-comparison of ‘Genome 1’ (using its paralogous genes). Parameters such as the minimum size and number of chromosomes to show can be further adjusted there.

KaryoView allows users to visualize a karyotype according to the colors of the syntenic chromosomes of a different genome. On Figure 4B, one can notice how human chromosomes map to the ancestral chromosomes of the boreoeutherian ancestor. The reconstructed boreoeutherian genome is consistent with previous studies (7) based on comparative fluorescent in situ hybridizations to infer eutherian ancestral karyotype (which is older than *Boreoeutheria* by only 5 million years). For example, the known fusion of two ancestral chromosomes (here blocks 25 and 27) leading to human chromosome 2 is represented, as well as the split of an ancestral block (here number 36) leading to chromosome 14 and 15 in *Homo sapiens*. Gene names of ‘Genome 2’ can be pasted in the text box to visualize the position of their orthologs or ancestors in ‘Genome 1’. Here, the four query genes are taken from the four human Hox gene clusters, and the red arrows on the karyotype show the position of the ancestral Hox gene clusters on the boreoeutherian genome.

Mouse-over functions and right-click menus render the KaryoView page very interactive. For instance, mouse-over will highlight chromosomes of interest and users may show or hide specific chromosomes from genome 1 by right-clicking on its name.

MatrixView is a more conventional dotplot of orthologous genes in two genomes, where coordinates are the relative positions of the genes in the two genomes being compared. Of interest to some users, duplications within a genome may be studied by plotting paralogs instead of the traditional orthologs. Here in Figure 4C, we represent a MatrixView of human genes plotted against their reconstructed boreoeutherian ancestor. Users can select specific part of the matrix and zoom to a chromosome of interest. Mouse-over and right-click utilities can be also used in this representation. For example, by clicking on the gene of interest in MatrixView, user will open the default PhyloView page with the selected gene as reference.

## GENOMICUS SOFTWARE IMPLEMENTATION

Genomicus is composed of Perl (version 5.8) scripts and modules, executed with mod_perl on an Apache2 (version 2.2) server and querying a MariaDB (version 5.5) database. The pages embed inline-SVG drawings in XHTML, while the JavaScript usage is limited to an information panel retrieved with AJAX calls. The interface is optimized for Firefox and Chrome navigators, but it also runs on Safari and Internet Explorer. The source codes of Genomicus and the MariaDB schema can be requested by email.

## FUTURE PLANS

At the present time, the major display improvements are available on the main Genomicus server dedicated to vertebrates. Future releases of GenomicusPlants, GenomicusMetazoa and GenomicusProtist will benefit from this new version of the code. Another perspective is to compute and represent regulatory interactions in other extant genomes (currently only human) and reconstructed ancestral genomes. We also plan to explore the possibility of representing ancestral transposable elements in their respective genomes, in order to extend the possibility to study the evolution of non-coding elements.
